# Investigation of Lactate, Base Excess, and Alactic Base Excess in Patients With Upper Gastrointestinal Bleeding

**DOI:** 10.1155/emmi/5750496

**Published:** 2025-10-22

**Authors:** Yilmaz Ersoz, Elmas Biberci Keskin, Basar Cander, Julide Yilmaz, Fatma Cakmak, Bahadir Taslidere

**Affiliations:** ^1^Department of Emergency Medicine, Faculty of Medicine, Bezmialem Vakif University, Istanbul, Türkiye; ^2^Department of Internal Medicine, Sisli Hamidiye Etfal Training and Research Hospital, Istanbul, Türkiye; ^3^Department of Emergency Medicine, Atlas University, Istanbul, Türkiye

**Keywords:** alactic base excess, base excess, upper gastrointestinal bleeding

## Abstract

**Introduction:**

Gastrointestinal system (GIS) bleeding is one of the most common reasons for emergency department visits. The aim of this study is to investigate the impact of base excess, lactate, and alactic base excess—parameters that can be quickly assessed through blood gas analysis—on predicting the clinical outcome in patients presenting to the emergency department with GIS bleeding.

**Materials and Methods:**

The study was retrospective and conducted at a single center from January 1 to December 31, 2022. The collected data included age, gender, blood gas (lactate and base excess), and outcomes (discharge, intensive care, and death).

**Results and Conclusions:**

The study included 205 patients (135 males and 70 females). Deceased individuals had a lower average base excess and higher lactate levels. There were no significant differences in ward admission based on alactic base excess status. A base excess cutoff value of ≥ −0.65 was used to predict ward admission, yielding a sensitivity of 57.71%, specificity of 63.33%, PPV of 90.18%, and negative predictive value of 20.43%. According to the data obtained in our study, we observed that a lactate cutoff value ≥ 2.07 could be effective in predicting ICU admission for patients. We also found that a base excess cutoff value ≥ −0.65 could be effective in predicting admission to the ward. The mean base excess was lower in patients who resulted in death compared to survivors, while the mean lactate level was higher. However, no significant result was found regarding alactic base excess.

## 1. Introduction

Upper gastrointestinal (GI) bleeding is one of the common reasons for emergency department visits and carries a significant risk of mortality and morbidity. Despite advancements in treatment protocols, mortality rates for upper GI bleeding remain around 10%. In the United States, the annual hospitalization rate due to upper GI bleeding is approximately 65 per 100,000 population. While it is more frequently observed in males, the incidence of the condition increases markedly, particularly in individuals over the age of 70 [1]. Rapid clinical decision making, accurate intervention, and risk stratification are crucial for patients presenting to the emergency department with GI bleeding. Various scoring systems and biochemical markers are utilized in this process to guide clinicians.

Lactate is a parameter frequently used to predict morbidity and mortality in critically ill patients. Elevated lactate levels are generally considered an indicator of poor prognosis and tissue hypoperfusion. A statistical relationship between elevated lactate levels and mortality in upper GI bleeding is well documented [2]. Base excess, another important parameter used to assess metabolic status, reflects the severity of hemodynamic disturbances. Recently, novel parameters such as alactic base excess (aBE) (the sum of base excess and lactate) have gained attention. The base excess, lactate, and aBE are thought to be effective in predicting mortality, particularly in patients experiencing shock [3].

This study aims to investigate the efficacy of parameters such as base excess, lactate, and aBE, which can be rapidly measured through blood gas analysis, in predicting the clinical course of patients presenting to the emergency department with upper GI bleeding.

## 2. Materials and Methods

This study was conducted observationally and retrospectively in the emergency department after obtaining ethical committee approval. Patients aged 18 years and older who presented to the emergency department and were diagnosed with upper GI bleeding were included in the study. Data from a 1-year period between January 1, 2022, and December 31, 2022, were retrospectively retrieved and analyzed from the hospital's automation system. Codes related to upper GI bleeding according to ICD-10 classification were identified. Exclusion criteria included patients referred from other centers with an established diagnosis, cases without bleeding originating from the upper GI tract, patients with esophageal variceal bleeding, trauma, pregnancy, malignancy, and those with incomplete data.

A comprehensive data collection form was prepared to include information on the patients' protocol numbers, age, sex, chronic diseases, and their severity, presenting complaints, symptoms (hematochezia, hematemesis, melena, syncope, and active bleeding), blood pressure, rectal examination findings, initial renal function tests (urea and creatinine), blood gas parameters (lactate and base excess), bleeding profile (prothrombin time [PT], activated partial thromboplastin time [aPTT], international normalized ratio [INR], hemoglobin, hematocrit, and platelet counts), transfusion information, endoscopy findings, whether rebleeding occurred during follow-up, complications developed during hospitalization, outcomes (discharge, death, and admission to the inpatient ward or intensive care unit [ICU]), and lengths of stay in the emergency department and hospital. In this study, lactate and base deficit values obtained from arterial blood gas analyses performed within the first 2 hours of admission to the emergency department were utilized. The lactate-based base excess (aBE) was calculated for each patient using the formula: “aBE (mmol/L) = Standard Base Excess (mmol/L) + Lactate (mmol/L).” According to aBE levels, patients were classified into three categories: low (< −3 mmol/L), moderate (−3-4 mmol/L), and high (≥ 4 mmol/L). The effects of base excess, lactate concentration, and aBE on prognosis prediction and mortality in patients with upper GI bleeding were analyzed. The criteria for ICU admission were defined as follows: hemodynamic instability (systolic blood pressure < 90 mmHg, tachycardia [> 100–110 bpm], and shock index > 1), need for vasopressor support, ongoing massive bleeding or presence of active hematemesis/melena, requirement of four or more units of erythrocyte suspension within 24 h, and a Glasgow-Blatchford Score (GBS) ≥ 12.

The study sample size was determined using a sample size calculation formula based on the total number of patients presenting to the emergency department within one year (400 patients). With a 95% confidence interval and a 5% margin of error, the minimum required sample size was calculated as 197, using a standard deviation of 0.5 and a Z-score of 1.96.

Statistical analysis: The data were analyzed using SPSS Version 25.0. The normality of the variables was assessed with histogram graphs and the Kolmogorov–Smirnov test. Descriptive statistics were presented as mean, standard deviation, median, and minimum-maximum values. For 2 × 2 comparisons, the Pearson chi-square test was used. Nonnormally distributed (nonparametric) variables were evaluated between groups using the Mann–Whitney *U* Test. Base excess and lactate threshold values were compared in binary groups and presented with sensitivity, specificity, positive predictive value (PPV), and negative predictive value (NPV). Additionally, ROC analysis was performed to determine the threshold values for base excess and lactate, and the curve was plotted. Binary logistic regression analysis was performed to evaluate whether base deficit and lactate levels were independent predictors of ICU admission and mortality. A *p* value of less than 0.05 was considered statistically significant.

## 3. Results

**Figure 1:**
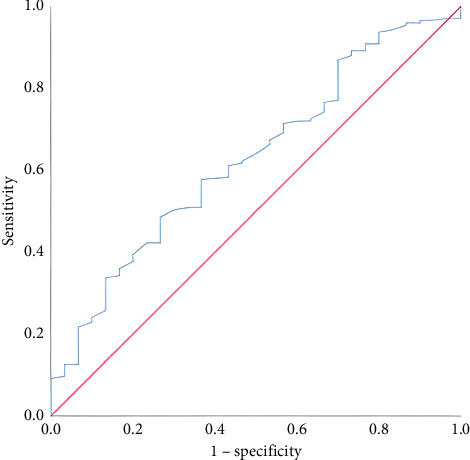
ROC curve analysis of base excess levels for predicting ward hospitalization.

**Figure 2:**
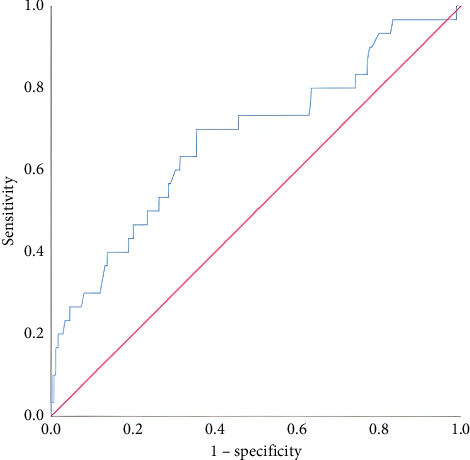
ROC curve analysis of lactate levels for predicting intensive care unit hospitalization.

A total of 205 patients were included in the study, consisting of 135 males (65.9%) and 70 females (34.1%). The median age of the participants was 69 years (range: 20–96). Regarding hospitalization status, 175 patients (85.4%) were admitted to the ward, while 30 patients (14.6%) were admitted to the ICU. The median length of hospital stay was 4 days (range: 1–37). Survival was achieved in 194 patients (94.6%), whereas 11 patients (5.4%) died. Of the 205 patients included in the study, 141 (68.8%) had comorbid conditions. The most common accompanying diseases were hypertension (*n* = 90, 43.9%), diabetes mellitus (*n* = 51, 24.8%), coronary artery disease (*n* = 51, 24.8%), heart failure (*n* = 23, 11.2%), kidney disease (*n* = 20, 9.7%), liver disease (*n* = 17, 8.3%), malignancy (*n* = 13, 6.3%), and cerebrovascular event (*n* = 6, 3%). Additionally, a history of previous GI bleeding was identified in 54 patients (26.4%). The presenting complaints of the patients included dyspepsia (*n* = 79, 38.5%), abdominal pain (*n* = 57, 27.8%), nausea/vomiting (*n* = 50, 24.3%), dizziness (*n* = 9, 4.3%), and syncope (*n* = 9, 4.3%). The endoscopic findings were as follows: duodenal ulcer (*n* = 62, 30.2%), gastric ulcer (*n* = 50, 24.3%), no visible lesion (*n* = 30, 14.4%), malignancy (*n* = 16, 7.8%), angiodysplasia (*n* = 14, 6.8%), erosive hyperemic gastritis (*n* = 11, 5.4%), ulcerative esophagitis (*n* = 9, 4.4%), and active bleeding (*n* = 9, 4.4%). Blood transfusion was required in 51 out of 205 patients (24.8%). Rebleeding was observed in 14 patients (6.8%). Shock, defined as systolic blood pressure below 90 mmHg accompanied by altered mental status, occurred in 23 patients (11.2%). The distribution of aBE was as follows: 12 patients had low levels, 138 had moderate levels, and 55 had high levels. Comparisons were made according to the type of hospitalization (Table 1). Patients admitted to the ICU had higher mean age, hospital stay duration, BUN, and lactate levels compared to those admitted to the ward. Conversely, their mean saturation, hemoglobin, hematocrit, erythrocyte, and base excess levels were lower. Survivors and nonsurvivors were compared in terms of hospital stay duration, base excess, lactate, and aBE levels (Table 2). In deceased patients, the mean base excess was lower, while the mean lactate level was higher than in survivors. The distribution of hospitalizations in the ward and ICU according to aBE groups is presented (Table 3). No significant differences were found between the groups. A significant cutoff value for predicting ward admission based on base excess levels was examined. When the cutoff value was set at ≥ −0.65 mmol/L, sensitivity was 57.71%, and specificity was 63.33% (Table 4; Figure 1). A significant cutoff value for predicting ICU admission based on lactate levels was also examined. When the cutoff value was set at ≥ 2.07 mmol/L, sensitivity was 63.33%, and specificity was 68.57% (Table 5; Figure 2). Logistic regression analysis identified lactate level as an independent and statistically significant predictor of ICU admission (Table 6). According to the logistic regression analysis, base excess was identified as an independent and statistically significant predictor of in-hospital mortality. This finding suggests that less negative base excess values are associated with a lower risk of death (Table 7).

## 4. Discussion

Acute upper GI bleeding is a life-threatening medical emergency that requires careful evaluation to reduce the risk of mortality. In our study, we examined the role of lactate, base excess, and aBE in predicting mortality and morbidity. The literature indicates that upper GI bleeding is most commonly observed in the elderly population, with age being an independent risk factor [4]. In our study, the mean age was 69 (20–96) years, consistent with the literature. The risk of bleeding increases with advanced age due to factors such as peptic ulcer risk and the use of medications. Male gender has been associated with a higher incidence of upper GI bleeding; in our study, the male proportion was 65.8%. This finding may be explained by lifestyle factors such as alcohol and smoking consumption. In the literature, hormonal and dietary differences in females are suggested to have protective effects [5, 6]. However, further research is needed to clarify the impact of gender on upper GI bleeding. In our study, no significant difference was observed between genders in terms of ward and ICU admission rates. These results align with the literature and highlight the significance of age and gender as key factors in upper GI bleeding [7].

Lactate levels are an important biomarker for predicting mortality and prognosis in patients with acute upper GI bleeding. In a 2019 study by Gulen et al., the mean venous lactate level was reported as 2.63 mmol/L, rising to 4.80 mmol/L in patients with fatal outcomes [8]. Various threshold values for lactate have been defined in the literature; for instance, El-Kersh et al. reported that a threshold of 2.1 mmol/L provided high sensitivity but low specificity [9]. Studies by Shrestha and Berger demonstrated that lactate levels > 2 mmol/L were associated with ICU admission and mortality [10]. In our study, a lactate level ≥ 2.07 mmol/L was found to be effective in predicting the need for ICU care. Lactate levels in deceased patients (2.16 [1.1 to 8.45] mmol/L) were significantly higher compared to survivors (1.78 [0.39 to 9.71] mmol/L). The literature further indicates that initial lactate levels ≥ 5 mmol/L are associated with markedly increased mortality. Additionally, Lee et al. reported that lactate clearance was correlated with 30-day recanalization and mortality rates. These findings suggest that lactate clearance may be more useful than lactate levels alone for patient monitoring [2]. In conclusion, lactate measurements serve as a valuable tool for risk assessment and management in patients with GI bleeding.

aBE, defined as the sum of lactate and standard base excess, has emerged as a useful parameter for evaluating acid-base imbalances. Gattinoni et al. highlighted the value of aBE in distinguishing lactate-driven acidosis from fixed acid retention in sepsis [11]. In a 2024 study by Hoque et al., negative aBE (< −3 mmol/L) was associated with early detection of renal dysfunction and high mortality in septic patients [12]. Smuszkiewicz's 2020 study identified a 28-day mortality increase in ICU patients with aBE values < −3.63 mmol/L [3]. Similarly, Cantos et al. reported in 2023 that the inability to normalize negative aBE was linked to increased mortality [13]. In our study, the mean aBE was 2.15 mmol/L, with values of 2.27 mmol/L in ward patients and 1.42 mmol/L in ICU patients; however, no statistical significance was observed. Among survivors, the mean aBE was 2.29 mmol/L, while it was −0.28 mmol/L in nonsurvivors. These findings underscore the need for more extensive studies on the use of aBE in patients with GI bleeding. Unlike sepsis-focused studies, the composition of our sample, which consisted of GI bleeding patients, may have contributed to differences in the results. Larger studies with more patients are warranted to better determine the clinical utility of aBE. Although hypovolemia develops in GI bleeding, aBE may not deteriorate as significantly as in septic shock. This may be due to the delayed onset of anaerobic metabolism, rapid improvement in perfusion through prompt fluid resuscitation, and the infrequent occurrence of associated organ dysfunction. Therefore, aBE may have limited value in reflecting the severity of shock in patients with GI bleeding.

Base excess has been defined as a marker for assessing the severity of metabolic acidosis or alkalosis independent of respiratory disorders. Values between −2 and +2 mmol/L are considered normal, with values below −2 indicating metabolic acidosis and values above +2 indicating metabolic alkalosis. In a 2015 study by Ko et al., the base excess was found to be −0.6 mmol/L in normotensive upper GI bleeding patients who later developed hypotension, compared to 1.5 mmol/L in those who remained normotensive, and this difference was statistically significant (*p* < 0.01) [14]. In a study by Lu et al., the base excess in patients with hemorrhagic shock was measured at −8.5 mmol/L, demonstrating significantly lower base excess levels in shock patients [15]. In our study, we found that base excess is a useful parameter for monitoring mortality and prognosis in patients with upper GI bleeding. Statistically significant differences in base excess values were observed between ward and ICU patients, as well as between survivors and nonsurvivors. Base excess emerges as a valuable biomarker for estimating and monitoring the degree of bleeding and tissue hypoperfusion. In our study, a base excess cutoff value of ≥ −0.65 mmol/L was identified to predict ward admission with a sensitivity of 57.71% and a specificity of 63.33%. Base excess was found to be lower in deceased patients (−2.3 [−14.7 to 6.5] mmol/L). These findings suggest that base excess is an important parameter for monitoring hemodynamic status and perfusion in patients with upper GI bleeding. Although aBE appeared to be a promising parameter in evaluating shock severity, no statistically significant difference was observed in our study. This discrepancy may be attributed to several factors, including sample size limitations, early intervention in GI bleeding cases, or differences in the underlying pathophysiological processes compared to septic shock. Further studies with larger populations and standardized timing of aBE measurements may help clarify its true clinical utility.

### 4.1. Limitation

The main limitations of this study include the risk of bias and missing data due to its retrospective design, limited generalizability as it was conducted in a single center, and the exclusion of specific patient groups, which may affect the applicability of the results to broader populations.

## 5. Conclusion

In our study, it was determined that lactate and base excess values could be effective in predicting clinical outcomes. The identified cutoff value for lactate (≥ 2.07 mmol/L) was found to be significant in predicting the need for ICU admission. This indicates that lactate may serve as a marker for tissue hypoxia and hemodynamic instability. The cutoff value for base excess (≥ −0.65 mmol/L) was found to be effective in predicting hospital ward admission. This finding suggests that base excess may be useful in assessing the severity of metabolic acidosis and predicting the prognosis of the disease. In patients who experienced fatal outcomes, the average base excess in our study was lower, while the average lactate level was higher compared to survivors, suggesting a potential association of these parameters with poor prognosis. Further studies with larger sample sizes are required to better clarify the clinical utility of parameters such as aBE.

## Figures and Tables

**Table 1:** Distribution of patients' vital signs, laboratory results, age, and gender according to hospitalization department.

Admission	Service	ICU	*p* value
Gender			
Male	118 (67.43%)	17 (56.67%)	0.251^∗^
Female	57 (32.57%)	13 (43.33%)	
Age	69 (20–93)	77.5 (49–96)	0.008
Length of stay	4 (1–37)	9.5 (1–31)	< 0.001
Fever	36.1 (35–39.2)	36.1 (35.2–37.8)	0.275
Pulse	91 (51–155)	97.5 (60–127)	0.794
Systolic BP	127 (67–261)	116.5 (73–187)	0.275
Diastolic BP	67.33 ± 17.66	65.13 ± 13.44 (40–89)	0.575
Saturation	97 (87–100)	96 (83–100)	0.021
MCV	87.8 (62.89–124.3)	90.25 (63.22–101.1)	0.110
Hemoglobin	9.85 (4.5–16.77)	8.49 (3.81–14.1)	0.030
Hematocrit	30.57 ± 8.55	26.73 ± 8.42	0.029
Platelets	224 (31–1245)	236 (17–469)	0.516
Erythrocytes	3.52 ± 1.01	3 ± 0.93	0.009
BUN	31 (7.94–120)	36.22 (10.28–187.38)	0.035
Creatinine	1.01 (0.47–14.4)	1.18 (0.7–5.21)	0.107
Sodium (Na)	138 (125–152)	135.5 (123–148)	0.067
Potassium (K)	4.34 (2.75–8.2)	4.31 (3.06–5.81)	0.691
HCO3	24 (14.5–36)	23.1 (17–29.6)	0.067
Base excess	0.5 (−14.7–17.4)	−1.45 (−8.5–5.1)	0.025
Lactate	1.75 (0.39–8.82)	2.29 (0.44–9.71)	0.002
Alactic base excess	2.4 (−11.73–19.23)	0.94 (−6.34–8.65)	0.222
pH	7.4 ± 0.05	7.4 ± 0.07	0.429

*Note:* HCO_3_: bicarbonate; pH: hydrogen ion concentration.

Abbreviations: BP = blood pressure, BUN = blood urea nitrogen, ICU = intensive care unit, MCV = mean corpuscular volume.

^∗^Chi-square test/Mann Whitney-*U* testi, *n*/mean ± SS, (min-max).

**Table 2:** Comparison of clinical parameters between 1 month survivors and nonsurvivors.

Outcome	Survived within 1 month	Death within 1 month	*p* value^∗^
Length of stay	4 (1–37)	4 (1–12)	0.818
Base excess	0.25 (−10.9–17.4)	−2.3 (−14.7–6.5)	0.027
Lactate	1.78 (0.39–9.71)	2.16 (1.1–8.45)	0.023
Alactic base excess	2.34 (−5.64–19.23)	0.81 (−11.73–8.65)	0.185

^∗^Chi-square test/Mann–Whitney *U* test; *n*/mean (min-max); *p* < 0.05 is considered statistically significant.

**Table 3:** Hospitalization distribution by alactic base excess status.

Admission	Service	ICU	*p* value
Alactic base excess			
Low	10 (5.71%)	2 (6.66%)	
Moderate	117 (66.86%)	21 (70%)	0.888
High	48 (27.43%)	7 (23.33%)	

*Note:* Chi-square test, Mann–Whitney *U* test, *n*/mean (min-max).

**Table 4:** ROC analysis of base deficit for predicting ward hospitalization.

	AUC	*p*	Cutoff	Sensitivity (%)	Specificity (%)	PPV (%)	NPV (%)
Service	0.628	0.025	≥ −0.65	57.71	63.33	90.18	20.43

*Note:* ROC analysis.

**Table 5:** ROC analysis of lactate for predicting ICU hospitalization.

	AUC	*p*	Cutoff	Sensitivity (%)	Specificity (%)	PPV (%)	NPV (%)
ICU	0.676	0.002	≥ 2.07	63.33	68.57	25.68	91.60

*Note:* ROC Analysis.

**Table 6:** Binary logistic regression analysis for prediction of ICU admission based on lactate and base deficit levels.

Variable	*B*	SE	Wald	*p*	OR (Exp(*B*))	95% CI for OR
Lactate	0.411	0.133	9.532	0.002	1.509	1.168–1.948
Base excess	−0.052	0.052	1.012	0.315	0.949	0.857–1.051
Constant	−2.842	0.378	56.457	< 0.001	—	—

**Table 7:** Binary logistic regression analysis for prediction of mortality based on lactate and base deficit levels.

Variable	*B*	SE	Wald	*p*	OR (Exp(*B*))	95% CI for OR
Lactate	0.272	0.154	3.115	0.078	1.313	0.970–1.776
Base excess	−0.170	0.082	4.346	0.038	0.844	0.719–0.990
Constant	−0.472	0.812	0.338	0.561	—	—

## Data Availability

The data supporting the findings of this study are available from the corresponding author upon reasonable request.

## References

[1] Wuerth B. A., Rockey D. C. (2018). Changing Epidemiology of Upper Gastrointestinal Hemorrhage in the Last Decade: A Nationwide Analysis. *Digestive Diseases and Sciences*.

[2] Lee S. H., Min Y. W., Bae J. (2017). Lactate Parameters Predict Clinical Outcomes in Patients With Nonvariceal Upper Gastrointestinal Bleeding. *Journal of Korean Medical Science*.

[3] Smuszkiewicz P., Jawień N., Szrama J., Lubarska M., Kusza K., Guzik P. (2022). Admission Lactate Concentration, Base Excess, and Alactic Base Excess Predict the 28-Day Inward Mortality in Shock Patients. *Journal of Clinical Medicine*.

[4] Lenti M. V., Pasina L., Cococcia S. (2019). Mortality Rate and Risk Factors for Gastrointestinal Bleeding in Elderly Patients. *European Journal of Internal Medicine*.

[5] Taşlidere B., Bíbercí Keskín E., Özdemír S., Atsiz A., Sönmez E. (2023). Comparison of Glasgow Blatchford and New Risk Scores to Predict Outcomes in Patients With Acute Upper GI Bleeding. *Bezmialem Science*.

[6] Heitkemper M. M., Chang L. (2009). Do Fluctuations in Ovarian Hormones Affect Gastrointestinal Symptoms in Women With Irritable Bowel Syndrome?. *Gender Medicine*.

[7] Essani Z. M. S., Naeem F., Parkash O. (2024). Risk Factors and Outcomes of Upper Gastrointestinal Bleeding in Hospitalized Patients in a Tertiary Care Hospital. *Journal of Pakistan Medical Association*.

[8] Gulen M., Satar S., Tas A., Avci A., Nazik H., Toptas Firat B. (2019). Lactate Level Predicts Mortality in Patients With Upper Gastrointestinal Bleeding. *Gastroenterology Research and Practice*.

[9] El-Kersh K., Chaddha U., Sinha R. S., Saad M., Guardiola J., Cavallazzi R. (2015). Predictive Role of Admission Lactate Level in Critically Ill Patients With Acute Upper Gastrointestinal Bleeding. *Journal of Emergency Medicine*.

[10] Shrestha M. P., Borgstrom M., Trowers E. A. (2018). Elevated Lactate Level Predicts Intensive Care Unit Admissions, Endoscopies and Transfusions in Patients With Acute Gastrointestinal Bleeding. *Clinical and Experimental Gastroenterology*.

[11] Gattinoni L., Vasques F., Camporota L. (2019). Understanding Lactatemia in Human Sepsis. Potential Impact for Early Management. *American Journal of Respiratory and Critical Care Medicine*.

[12] Hoque M., Nagourney J., Pawlowski T. (2024). Alactic Base Excess (ABE): A Novel Internal Milieu Parameter-Its Concept and Clinical Importance. *International Urology and Nephrology*.

[13] Cantos J., Huespe I. A., Sinner J. F. (2023). Alactic Base Excess is an Independent Predictor of Death in Sepsis: A Propensity Score Analysis. *Journal of Critical Care*.

[14] Ko B. S., Kim W. Y., Ryoo S. M. (2015). Predicting the Occurrence of Hypotension in Stable Patients With Nonvariceal Upper Gastrointestinal Bleeding: Point-of-Care Lactate Testing. *Critical Care Medicine*.

[15] Lu B., Li M. Q., Li J. Q. (2015). The Use of Limited Fluid Resuscitation and Blood Pressure-Controlling Drugs in the Treatment of Acute Upper Gastrointestinal Hemorrhage Concomitant With Hemorrhagic Shock. *Cell Biochemistry and Biophysics*.

